# Adenoma of the Papillae of Vater. Report of Eleven Cases

**DOI:** 10.1155/2000/91250

**Published:** 2000-01

**Authors:** Heikki Kiviniemi, Jyrki Mäkelä, Jukka Palm, Arto Saarela

**Affiliations:** 1 Department of Surgery Oulu University Hospital Oulu Finland; 2 Department of Surgery Division of Gastroenterology Oulu University Hospital Kajaanintie 50 Oulu FIN-90220 Finland

## Abstract

Eleven patients with a preoperative diagnosis of adenoma of the papillae of Vater were followed up during the fifteen-year period from 1984 till 1998 in the Oulu University Hospital. Seven patients were treated primarily by transduodenal excision without any recurrences so far. One of these seven patients was found to have adenocarcinoma in a histological examination.

Active surgery for adenoma of the papillae of Vater is recommended because of the precancerous nature of the lesion, and because malignancy cannot always be detected by endoscopic biopsies. Transduodenal excision could be recommend for patients at high operative risk, especially in cases with small adenomas and low-grade dysplasia, where histologically free resection margins can be achieved, but pancreaticoduodenectomy should still be performed on patients at low operative risk.


*HPB Surgery*, 2000, Vol. 11, pp. 339-344
Reprints available directly from the publisher
Photocopying permitted by license only

© 2000 OPA (Overseas Publishers Association) N.V.
Published by license under
the Harwood Academic Publishers imprint,
part of The Gordon and Breach Publishing Group.
Printed in Malaysia.

*Case Report*

### Adenoma of the Papillae of Vater. Report of Eleven Cases

HEIKKI KIVINIEMI*, JYRKI MÄKELÄ, JUKKA PALM and ARTO SAARELA

*Department of Surgery, Oulu University Hospital, Oulu, Finland*

*(Received 25 April 1998)*

**Eleven patients with a preoperative diagnosis of adenoma of the papillae of Vater were followed up during the fifteen-year period from 1984 till 1998 in the Oulu University Hospital. Seven patients were treated primarily by transduodenal excision without any recurrences so far. One of these seven patients was found to have adenocarcinoma in a histological examination.**

**Active surgery for adenoma of the papillae of Vater is recommended because of the precancerous nature of the lesion, and because malignancy cannot always be detected by endoscopic biopsies. Transduodenal excision could be recommend for patients at high operative risk, especially in cases with small adenomas and low-grade dysplasia, where histologically free resection margins can be achieved, but pancreaticoduodenectomy should still be performed on patients at low operative risk.**

*Keywords:* Adenoma, papilla of Vater, transduodenal excision, pancreaticoduodenectomy

### INTRODUCTION

True villous adenomas of the papillae of Vater are rare. It was as late as 1895 that Calzavara first described a benign ampullary papilloma of the papillae of Vater [1]. The clinical diagnosis of ampullary adenoma was uncommon before 1973, since which time ERCP has become widely used in the diagnosis of pancreaticobiliary diseases. With the increasing use of ERCP, adenomas of the papilla have been detected more often by a lateral duodenoscopy [2, 3].

The differentiation between a benign adenoma and an infiltrating carcinoma based on endoscopic specimens may be difficult [4]. It has also become evident that adenomas of the papilla are precancerous lesions [4-6]. The first transduodenal excision of an ampullary tumour was performed by Halsted in 1897 [7]. After that, local excision became the most common procedure for the treatment of ampullary tumours, with at least 85 cases reported by 1935 [8].

When Whipple introduced pancreaticoduodenectomy for the treatment of pancreatic and periampullary tumours in 1935, local excision was relegated to the background. Recently, a new

*Address for correspondence: Department of Surgery, Division of Gastroenterology, Oulu University Hospital, Kajaanintie 50, FIN-90220 Oulu, Finland.

interest in the local treatment of ampullary lesions has arisen, and it has been suggested that ampullary excision is still useful [9]. Eleven such patients have been treated in the Department of Surgery of the Oulu University Hospital during the past fifteen years, and seven of them have been diagnosed and treated during the last four years. These patients constituted the series reported here.

### MATERIAL AND METHODS

All patients subjected to endoscopic or surgical treatment of ampullary adenoma in the Oulu University Hospital between 1984 and 1998 were retrospectively reviewed. The symptoms and laboratory findings at admission were recorded. Ultrasonography was made in all cases and computer tomography on two patients. ERCP with endoscopic papillotomy and biopsy was a routine. The operative findings, the operative treatment methods and the final histology of the tumour were recorded. Postoperative complications and follow-up data were noted as well.

### RESULTS

The clinical findings at presentation and the laboratory findings on liver and pancreas chemistry obtained immediately after admission are listed in Table I. Epigastric colic (7/11) with or without icterus was the most common clinical symptom on admission, and two patients presented with recurrent acute pancreatitis. Elevated liver chemistry values were the most common laboratory finding.

Ultrasonography did not reveal the tumour in any of our cases. Dilatation of the intra or extrahepatic bile ducts was seen in five patients. Computer tomography, which was performed on two patients, showed a papillary tumour of one centimeter in diameter in the distal bile duct in the first case, but was negative in the other case. Endoscopic retrograde cholangiopancreaticography was performed in all cases. Biopsy showed five tubular and six tubulovillous adenomas with moderate dysplasia in all cases (Tab. II).

TABLE I Clinical and laboratory findings on admission

| Case | Age | Clinical finding | Laboratory finding |
|---|---|---|---|
| 1 | 64 | Recurrent pancreatitis | Normal liver chemistry |
| 2 | 70 | Colic | Elevated liver chemistry |
| 3 | 41 | Recurrent pancreatitis | Hyperamylasemia |
| 4 | 61 | Colic | Elevated liver chemistry |
| 5 | 77 | Colic | Normal liver chemistry |
| 6 | 71 | Colic | Elevated liver chemistry |
| 7 | 64 | Colic and Periodic icterus | Elevated liver chemistry |
| 8 | 35 | Colic | Hyperamylasemia |
| 9 | 81 | Icterus | Elevated liver chemistry |
| 10 | 63 | No symptoms | Anemia |
| 11 | 81 | Colic and Icterus | Elevated liver chemistry |

Two patients with narrow stalked adenomas were treated by snare fulguration of the adenoma. Both of these patients developed a recurrence during follow-up. The first of them has been treated once and the other twice by transduodenal excision.

One patient (81-year-old male with severe aortic stenosis) was followed up with repeated endoscopies, and biliary drainage was secured by biliary endoprosthesis. Endoscopic biopsy showed repeatedly a tubular adenoma with moderate dysplasia. At the end of 1996, increased abdominal pain began, acute cholecystitis developed and the gallbladder was removed at laparotomy. No infiltrating tumour was detected during cholecystectomy, but severe inflammation of the hepatoduodenal ligament made the diagnosis very difficult. The patient's condition improved for a while, but deteriorated again, ascites developed and he succumbed in January 1997. Autopsy showed adenocarcinoma of the distal choledochus and hepatic metastases.

Seven patients primarily and nine altogether underwent transduodenal excision without any significant postoperative complications. One patient developed two recurrences after the primary snare fulguration and underwent two

TABLE II Roentgenological findings and results of histological examination

| Case | Ultrasound | Computed tomography | ERCP | Biopsy |
|---|---|---|---|---|
| 1 | Septum in gallbladder | – | Suspicion of ampullary tumour | Tubular adenoma Moderate dysplasia |
| 2 | Gallbladder removed | – | 2–3 cm ampullary tumour | Tubular adenoma Moderate dysplasia |
| 3 | Normal | – | 0.5 cm ampullary tumour | Tubular adenoma Moderate dysplasia |
| 4 | Gallbladder removed | – | Defect in the distal choledochus | Tubulovillous adenoma Mild dysplasia |
| 5 | Dilated intrahepatic bile ducts | – | Ampullary tumour | Tubulovillous adenoma Moderate dysplasia |
| 6 | Dilated intrahepatic bile ducts | – | Ampullary tumour | Tubular adenoma Moderate dysplasia |
| 7 | Dilated intrahepatic bile ducts | – | Ampullary tumour | Tubulovillous adenoma Mild dysplasia |
| 8 | Normal | – | Ampullary tumour | Tubulovillous adenoma Mild dysplasia |
| 9 | Dilated intrahepatic bile ducts | – | Suspicion of ampullary tumour | Tubulovillous adenoma Moderate dysplasia |
| 10 | Normal | – | Ampullary tumour | Tubulovillous adenoma Moderate dysplasia |
| 11 | Dilated intrahepatic bile ducts | No tumour | Ampullary tumour | Tubular adenoma Mild dysplasia |

TABLE III Operative findings and results of postoperative histological examination

| Case | Operative finding | Frozen section | Final histology |
|---|---|---|---|
| 1 | 1 cm polypoid tumour | Yes | Tubulovillous adenoma Mild dysplasia |
| 2 | 2.5 cm stalky tumour | Yes | Tubulovillous adenoma Mild dysplasia |
| 3 | 1 cm polypoid tumour | Yes | Tubulovillous adenoma Moderate adenoma |
| 4 | 1.3 cm solid tumour | No | Tubulovillous adenoma Moderate dysplasia |
| 5 | 0.5 cm solid tumour | No | Tubulovillous adenoma Mild dysplasia |
| 6 | 1.5 cm polypoid tumour | No | Tubular adenoma Moderate adenoma |
| 7 | 4 cm sessile tumour | No | Adenocarcinoma 3 mm free margin |
| 8 | 3 cm solid tumour | No | Tubulovillous adenoma Mild dysplasia |
| 9 | Not operated | No | Tubulovillous adenoma Hepatic metastases |
| 10 | 1 cm solid tumour | No | Tubulovillous adenoma Severe dysplasia |
| 11 | Not operated | No | Tubulovillous adenoma Moderate dysplasia |

repeated local excisions. The final histology showed benign tubular adenoma with moderate dysplasia.

One patient underwent primary pancreaticoduodenectomy, but no operations have been converted to pancreaticoduodenectomy later. One adenocarcinoma was found in a postoperative histological examination of the specimen (Tab. III), but the tumour was radically removed by local excision with microscopically clear operative margins. We are not planning to perform a pancreaticoduodenectomy, because the patient is at increased operative risk due to cirrhosis of the liver and chronic obstructive lung disease. The follow-up has been intense with repeated endoscopies at three-month intervals.

Each of the last four patients had an endocoil prosthesis inserted into the bile duct before the operation. One of these patients also had the pancreatic duct cannulated. 100 mg of Octreotide administered subcutaneously three times a day was started preoperatively for prophylaxis against pancreatitis in all these patients. No cases of acute pancreatitis were recorded (Tab. IV).

### DISCUSSION

Although the clinical symptoms may suggest the diagnosis of adenoma of the ampulla of Vater, duodenoscopy with concomitant ERCP is the diagnostic procedure of choice. Ampullary tumours are easily identifiable in most cases, but up to 40% of the tumours occur in the intraampullary region [10], and these tumours are only visualized and biopsy specimens can only be taken after papillotomy [11]. Two of our patients had a smooth-lined defect in the distal choledochus, and both of these tumours were only visualized after papillotomy.

After ERCP was introduced on a large scale, increasing numbers of adenomas of the papillae

TABLE IV Surgical treatment, postoperative complications and follow-up data

| Case | TDE* | Complications | Follow-up data |
|---|---|---|---|
| 1 | Yes<br>T-drain into the pancreatic duct | No | 12 years |
| 2 | Yes<br>T-drain into the common bile duct | No | 12 years |
| 3 | Yes<br>Cholecystectomy | No | 8 years |
| 4 | Snare fulguration<br>TDE<br>Re-TDE | <br>Wound infection<br>Recurrence stenosis duodeni | <br>12 months |
| 5 | Yes<br>Preoperative stenting of bile and pancreatic duct | No | 15 months |
| 6 | Yes<br>Preoperative stenting of bile duct | No | 4 months |
| 7 | Yes<br>Preoperative stenting of bile duct | Respiratory insufficiency | 4 months |
| 8 | Pancreaticoduodenectomy | No | 6 years |
| 9 | Not operated<br>Repeated endoscopies | – | Died after one year follow-up |
| 10 | Yes<br>Preoperative stenting of bile duct | No | 3 months |
| 11 | Not operated<br>Snare fulguration | – | Recurrence after 18-month follow-up<br>Scheduled for TDE |

* Transduodenal excision.

of Vater have been diagnosed. Our patients were mostly diagnosed in the 1990s, while only two cases were treated in the 1980s.

There is significant evidence to suggest that adenoma of the papillae of Vater is a precancerous lesion [4, 5] similarly to adenoma of the colon and should be treated accordingly. Endoscopic biopsy of adenomas is unreliable in showing the carcinomatous foci in an adenomatous tumour [12]. The malignant potential of these adenomas was shown by one of our own patients, who was treated conservatively (81-year-old male with aortic stenosis). He was followed up with repeated endoscopic biopsies, and endoprosthesis was used to treat bile and pancreatic duct stenosis. In spite of intense local follow-up, adenocarcinoma of the distal choledochus with hepatic metastases developed one and a half years after the diagnosis, and the patient needed urgent surgery for acute cholecystitis. The unreliability of endoscopic biopsies in detecting malignancy was shown by one female patient subjected to extensive local excision : Histological specimens showed in this tumour a 2-cm-wide malignant lesion, which, however, was removed with clear operative margins.

The adenoma of the papillae Vater presents also a therapeutic dilemma. While there is a uniform agreement that these lesions should be resected, opinions differ as to the method of resection, ranging from endoscopic treatment [13] to radical pancreaticoduodenectomy [14–16]. The local excision, although generally associated with lower morbidity has a higher recurrence rate [13, 17–19]. The recurrence can occur with or without malignant transformation [20]. The recurrence rate must be weighted against the potential operative mortality and morbidity rates for pancreaticoduodenectomy. According to recent series [2, 11, 21] as well as our own series the transduodenal excision is safe and could be ideal in selected patients with high operative risk for pancreaticoduodenectomy.

During the past decade several reports have advocated local ampullary resection also for selected patients with malignant lesions [12]. Although the comparison of local excision and pancreaticoduodenectomy is impossible because of selection bias [12, 17], excellent results with long-term survivors after local excision for cancer have been reported [18, 22–25].

The technique of transduodenal excision of ampullary tumours has received little attention [9, 11]. According to our experience, preoperative cannulation of the bile duct and if possible pancreatic duct, helps us to localize the walls of these ducts at operation. The possibility to excise the mucosa against the prosthesis is also a definite advantage. Unfortunately technical difficulties caused by the tumour often prevents cannulation. The localization of the pancreatic duct is often difficult during operation. Secretin can be used to stimulate pancreatic secretion to help to identify the pancreatic duct orifice [9]. The reconstruction is critical to ensure adequate biliary and pancreatic drainage and to prepare the transduodenal defect. Loupe magnification is rather useful for accurate reconstruction [19] and our experience in two last patients confirm this. T-tube drainage through the choledochotomy or stents through the anastomosis to the duodenum have been used but their usage is obviously not obligatory.

Two of our patients have been followed for 12 and 13 years, respectively, without any recurrence. In two of our patients the initial treatment was snare fulguration, but a recurrence developed in both cases within a year. For this reason we have abondoned this approach in favour of local transduodenal excision. The treatment of recurrent disease can be problematic. Some patients with benign small recurrences can be treated by local excision [19], but pancreaticoduodenectomy should be considered whenever possible. A re-excision was necessary for one of our patients 18 months after the primary operation. The re-excision lead to narrowing of the duodenum, requiring a gastrojejunostomy which should be considered as a prophylactic procedure, if a local excision is chosen for reoperation instead of pancreaticoduodenectomy.

In conclusion, transduodenal excision of ampullary tumour is technically safe and indicated in selected patients with high operative risk. The recurrence rate needs to be evaluated after longer follow-up.

### References

[1] Calzavara, C. (1895). Uber adenome des Verdanungskanals. *Wirchows Arch. (A)*, **141**, 221.

[2] Sand, J. A. and Nordback, I. M. (1995). Transduodenal excision of benign adenoma of the papilla of Vater. *Eur. J. Surg.*, **161**, 269–272.

[3] Sivak, M. V. (1988). Clinical and endoscopic aspects of tumours of the ampulla of Vater. *Endoscopy*, **20**, 211–217.

[4] Yamaguchi, K. and Enjoji, M. (1991). Adenoma of the ampulla of Vater: putative precancerous lesions. GUT, **32**, 1558–1561.

[5] Seifert, E., Schulte, F. and Stolte, M. (1992). Adenoma and carcinoma of the duodenum and papilla of Vater: a clinicopathologic study. *Am. J. Gastroenterol*, **87**, 37–42.

[6] Stolte, M. and Pscherer, C. (1996). Adenoma-carcinoma sequence in the papilla of Vater. *Scand J. Gastroenterol*, **31**, 376–382.

[7] Halsted, W. (1899). Contributions to the surgery of the bile passages, especially of the common bile duct. *Boston Med. Surg. J.*, **141**, 645–654.

[8] Hunt, V. C. and Budd, J. W. (1935). Transduodenal resection of the ampulla of Vater for carcinoma of the distal end of the common bile duct. *Surg. Gynecol. Obstet.*, **61**, 651–661.

[9] Kahn, M. B. and Rush, J. W. (1989). The overlooked technique of ampullary excision. *Surg. Gynecol. Obstet.*, **169**, 253–354.

[10] Ponchon, T., Berger, F., Chavaillon, A., Bory, R. and Lambert, R. (1989). Contribution of endoscopy to diagnosis and treatment of tumours of the ampulla of Vater. *Cancer*, **64**, 161–167.

[11] Alstrup, N., Burcharcth, F., Honge, C. and Horn, T. (1996). Transduodenal excision of tumours of the ampulla of Vater. *Eur. J. Surg.*, **162**, 961–967.

[12] Cahen, D. L., Fockens, P., deWit, L. Th., Offerhaust, G. J. A., Obertop, H. and Gouma, D. J. (1997). Local resection or pancreaticoduodenectomy for villous adenoma of the ampulla of Vater diagnosed before operation. *Br. J. Surg.*, **84**, 948–951.

[13] Shemesh, E., Nass, S. and Czerniac, A. (1989). Endoscopic sphincterotomy and endoscopic fulguration in the management of adenoma of the papilla of Vater. *Surg. Gyn. Obst.*, **169**, 445–448.

[14] Sobol, S. and Cooperman, A. M. (1978). Villous adenoma of the ampulla of Vater. An unusual cause of biliary colic and obstructive jaundice. *Gastroenterology*, **75**, 107–109.

[15] Oh, C. and Jemerin, E. (1965). Benign adenomatous polyps of the papilla of Vater. *Surgery*, **57**, 495–503.

[16] Schulten, M. F., Oyasu, R. and Beal, J. M. (1976). Villous adenoma of the duodenum. A case report and a review of literature. *Am. J. Surg.*, **132**, 90–96.

[17] Cray, G. and Browner, W. (1989). Villous tumors of the ampulla of Vater: local resection versus pancreaticoduodenectomy. *South Med. J.*, **82**, 917–920.

[18] Gerth, P. H., Matthews, J. B., Lerut, J., Baer, H. U. and Blumgart, L. H. (1990). The technique of papilloduodenectomy. *Surg. Gynecol. Obstet.*, **170**, 254–256.

[19] Branum, G. D., Panpas, T. N. and Meyers, W. C. (1996). The management of tumors of the ampulla of Vater by local excision. *Ann. Surg.*, **224**, 621–627.

[20] Lukes, P. J., Nilson, A. E., Rolny, P. and Gamblou, R. (1979). Premalignant lesions of the papilla of Vater and the common bile duct. *Acta Chir. Scand.*, **145**, 545–548.

[21] Farouk, M., Niotis, M., Branum, G. D., Cotton, P. B. and Meyers, W. C. (1991). Indications for and technique of local resection of tumours of the papilla of Vater. *Arch. Surg.*, **126**, 650–652.

[22] Rosenberg, J., Welch, J. P., Pyrtek, L. J., Walker, M. and Trowbridge, P. (1986). Benign villous adenomas of the ampulla of Vater. *Cancer*, **58**, 1563–1568.

[23] Goldberg, M., Zamir, O., Hadary, A. and Nissan, S. (1987). Wide local excision as an alternative treatment for periampullary carcinoma. *Am. J. Gastroenterol*, **82**, 1169–1171.

[24] Eggink, W. F., van Berge, Henegouwen, C. P., Brandt, K. H., Bronjhorst, F. B. and van der Heyde, M. N. (1988). Tumor of the ampulla of Vater treated by local resection: a report of five cases. *Neth. J. Surg.*, **40**, 110–113.

[25] Tarazi, R. Y., Hermann, R. E., Vogt, D. P. *et al.* (1986). Results of surgical treatment of periampullary tumors: a 35-years experience. *Surgery*, **100**, 716–723.

